# Dual legacy of plant water-deficit-tolerance and watering history shapes rhizosphere microbial transplant outcomes in tomato post-drought recovery

**DOI:** 10.1186/s40793-026-00953-0

**Published:** 2026-09-08

**Authors:** Rodrigo Pulgar, Alexis Gaete, Madelaine Mejías, Romina Madrid, Ignacio Chávez, Lucas Pino, Andrea Talamilla-Espinoza, Jeimy Moscote, Claudio Pastenes, Dinka Mandakovic

**Affiliations:** 1 Laboratorio de Genómica y Genética de Interacciones Biológicas, Instituto de Nutrición y Tecnología de los Alimentos, Universidad de Chile, El Líbano 5524, Santiago, 7810000 Chile; 2 Instituto Milenio Centro de Regulación del Genoma, Santiago, 7800003 Chile; 3 Laboratorio de Desarrollo Vegetal y Biotecnología, Facultad de Ciencias Biológicas, Pontificia Universidad Católica de Chile, Avenida Libertador Bernardo O’Higgins 340, Santiago, 8331150 Chile; 4 GEMA Center for Genomics, Ecology and Environment, Universidad Mayor, Camino La Pirámide 5750, Santiago, 8580745 Chile; 5 Programa de Doctorado en Ecología Integrativa, Universidad Mayor, Camino La Pirámide 5750, Santiago, 8580745 Chile; 6 Programa de Doctorado en Genómica Integrativa, Universidad Mayor, Camino La Pirámide 5750, Santiago, 8580745 Chile; 7 Programa de Doctorado en Ciencias Silvoagropecuarias y Veterinarias, Campus Sur Universidad de Chile, Santa Rosa 11315, Santiago, 8820808 Chile; 8 ANID–Millennium Scientific Initiative–Millennium Institute for Integrative Biology (iBIO), Santiago, Chile; 9 Center for Genomics and Bioinformatics, Universidad Mayor, Camino La Pirámide 5750, Santiago, 8580745 Chile; 10 Facultad de Ciencias Agronómicas, Universidad de Chile, Santa Rosa 11315, Santiago, 8820808 Chile

**Keywords:** Community legacy, Drought tolerance, Microbial inoculation, Microbial networks, Plant-microbial interactions, Rhizosphere microbiome

## Abstract

**Background:**

Climate change is intensifying drought events, negatively affecting crop yields and quality. Enhancing plant recovery following drought is therefore critical for agricultural resilience. Within the holobiont framework, the plant microbiome plays a key role in modulating plant responses to environmental stress. Here, we evaluated the contribution of rhizosphere microbial transplants derived from *Solanum lycopersicum* cultivars differing in water-deficit-tolerance and prior exposure or not to water deficit to post-drought recovery in susceptible tomato plants.

**Results:**

Physiological measurements, including net photosynthetic rate, relative water content, and water potential, showed that microbial community transplants from water-deficit-tolerant cultivars previously exposed to water deficit significantly enhanced post-drought recovery in susceptible plants, suggesting a dual legacy effect of host phenotype and irrigation history. High-throughput metabarcoding revealed marked temporal shifts in rhizosphere microbial communities between the initial (Ti) and post-recovery (Tf) stages. Bacterial alpha diversity increased significantly at Tf only for the tolerant inoculant under deficit irrigation, whereas fungal diversity showed more stable response across conditions. Beta-diversity analyses revealed clear separation between Ti and Tf communities for both bacteria and fungi. Community structure changes were highly pronounced in the inoculation treatments that produced the most contrasting post-drought recovery phenotypes (SFI: susceptible cultivar under full irrigation and TDI: tolerant cultivar under deficit irrigation), which formed distinct clusters in hierarchical analyses. Differential abundance analysis identified bacterial taxa enriched at Tf in TDI, while fungal communities displayed stage-specific markers. Functional annotation using FAPROTAX and FUNGuild revealed enrichment of nitrate reduction and anoxygenic photoautotrophy among bacterial functions, and saprotrophic guilds among fungal communities at Tf in TDI, suggesting increased nutrient cycling activity associated with post-drought recovery. Co-occurrence network analysis revealed contrasting interaction patterns, with higher connectivity in SFI and a greater contribution of fungal taxa and positive fungi-fungi interactions in TDI.

**Conclusions:**

These results indicate that the legacy of rhizosphere microbiomes shaped by host water-deficit-tolerance and irrigation history is associated with enhanced post-drought recovery, involving the enrichment of specific microbial taxa and functional guilds. The concept of dual legacy provides a useful framework for understanding microbiome-mediated plant resilience.

**Supplementary Information:**

The online version contains supplementary material available at https://doi.org/10.1186/s40793-026-00953-0.

## Background

Environmental stressors associated with climate change are increasingly threatening crop productivity worldwide, with negative impacts on both yield and nutritional quality of agronomically important species [1, 2]. Among these stressors, drought has become more frequent and severe, causing substantial economic losses, estimated at US$307 billion per year [3], and posing a major challenge to global food security [4]. Plant performance under drought conditions depends not only on the ability to tolerate water deficit but also on the capacity to recover after stress release. Indeed, post-drought recovery has emerged as a critical determinant of drought resilience and yield stability [5].

Projections indicate that by 2050, drought will critically threaten global crop production, with over 60 countries projected to experience drought-induced production losses exceeding 10% [4, 6, 7], driving the development of mitigation strategies such as crop rotation [8, 9], breeding and selection of drought-adapted cultivars [10–12], and the use of beneficial microorganisms to enhance plant stress tolerance [13, 14]. This latter strategy is grounded in the “holobiont” concept, which considers plants and their associated microbiota as an integrated functional unit. Within this framework, microbial communities form intimate associations with their hosts and contribute traits that can enhance plant performance under adverse environmental conditions [15, 16].

Among plant growth-promoting (PGP) microorganisms, taxa capable of nitrogen fixation [17], inorganic phosphate solubilization [18], and the production of siderophores [19], phytohormones [20], or enzymes that modulate senescence-related signaling pathways [21] have been widely described. These microorganisms have been applied as inoculants to mitigate various abiotic stresses, including drought [22–24]. However, despite their broad adoption, inoculation strategies based on single strains with in vitro PGP traits frequently fail under greenhouse and field conditions. This limitation likely arises because simplified inoculants fail to reproduce the complex plant-microbe and microbe-microbe interactions found in natural systems and because introduced strains often struggle to establish and persist when competing with native soil microbiota [25–27]. To overcome these limitations, synthetic microbial communities (SynComs) have been developed to reconstruct simplified yet controlled microbiomes by co-culturing multiple microbial taxa [28].

Although SynComs have advanced our mechanistic understanding of microbiome function, they do not always perform consistently, as they remain simplified microbial assemblages whose establishment and persistence also depend on competitive interactions with resident microbiota and successful rhizosphere colonization [29, 30]. Moreover, the outcome of SynCom inoculation is modulated by characteristics such as consortium composition, diversity, and inoculant density, as well as application strategies, highlighting the need for careful optimization before field deployment [26]. An alternative and increasingly promising approach is the use of naturally assembled plant-derived microbiota through whole-community microbial transplants. This strategy preserves a broader spectrum of microbial diversity and ecological interactions, including uncultivable taxa that may constitute the majority of environmental microorganisms [31]. Consistent with this approach, several studies have shown that microbial transplants that use drought to reshape the rhizosphere microbiota can enhance drought tolerance and post-drought recovery in subsequent plant generations [32–35]. Whole-rhizosphere transplants have thus been proposed as an effective means of transferring a microbial genetic pool, and its associated functional potential, from one host to another, contributing to improved plant stress resilience [36].

Because host strongly influences rhizosphere microbiome assembly [37–39], and environmental conditions further shape these microbial communities, this study aimed to evaluate the contribution of rhizosphere microbial transplants derived from thoroughly selected water-deficit-tolerant and susceptible *Solanum lycopersicum* cultivars, exposed or not to water deficit, to the post-drought recovery of susceptible tomato plants. We previously conducted an in-depth characterization of the rhizosphere microbiomes associated with these contrasting cultivars, showing that the surrounding microbiota may condition plant-water-deficit-related phenotypes [40]. Here, we hypothesize that rhizosphere microbial transplants derived from a water-deficit-tolerant cultivar exposed to water deficit will most effectively promote post-drought recovery in susceptible plants, driven by the combined legacy effects of plant water-deficit-tolerance and watering history on rhizosphere bacterial and fungal community composition and interactions.

## Methods

### Rhizosphere microbial transplants and plant tolerance measurements

The cultivation assay consisted of inoculating seeds from a water-deficit-susceptible *Solanum lycopersicum* cv. Advantage (US Department of Agriculture Database #PI600926) with rhizosphere microbial communities previously obtained and characterized in Gaete et al. (2021) [40]. These rhizospheres were derived from both a water-deficit-tolerant *Solanum lycopersicum* cv. 449 (US Department of Agriculture Database #PI128608) and the susceptible cv. Advantage, each subjected or not to water deficit in the original study. Accordingly, four distinct rhizosphere microbial community donors were used: susceptible cultivar under full irrigation (SFI), susceptible cultivar under deficit irrigation (SDI), tolerant cultivar under full irrigation (TFI), and tolerant cultivar under deficit irrigation (TDI). These microbial communities were used as inoculants for susceptible tomato seeds to assess plant post-drought recovery.

Throughout this manuscript, the term “water-deficit tolerance” is used to describe the phenotypic classification of the donor cultivars as established in Gaete et al. (2021) [40], where plants were characterized under controlled water-deficit conditions. The term “drought” refers specifically to the stress treatment imposed on recipient plants in the present study.

Donor rhizospheres were retrieved and processed as described in Gaete et al. (2021) [40]. Briefly, rhizosphere samples consisted of soil tightly adhering to the root surface after gently shaking the root systems to remove loosely attached soil. Roots were cut and washed in a 10 mM NaCl solution [41, 42]. Within 2–3 h after collection, the rhizosphere microbial communities were used to inoculate twelve surface-sterilized susceptible tomato seeds. Inoculants were prepared by mixing 5 g of the donor rhizospheres with 5 mL of sterile water at 300 rpm for 20 min at room temperature (RT), which were subsequently used for seed dipping during 2 h at RT. After sowing the inoculated seeds, the 5 mL of slurry donor rhizospheres were used to irrigate the sterile soils for each condition. The soil used for plant cultivation corresponded to the same substrate previously described by Gaete et al. (2021) [40]. It was a local semi-arid soil collected in Las Cardas, Coquimbo Region, Chile (30°15’07.6"S 71°15’24.2"W), in the transition zone toward the Limarí Valley, classified as a sandy loam soil derived from marine sediments of the “tambillo” series (typic halodurid) and containing less than 1% organic matter. The semi-arid soil was selected because it represented the original environment from which the donor rhizosphere microbial communities were obtained. Thus, microbial inoculants originated from rhizosphere communities assembled under low water availability conditions, and the same soil, after sterilization, was used as the substrate in the transplant experiment. This soil was sterilized by three consecutive autoclaves with a 48 h resting period between cycles. The effectiveness of soil sterilization was verified by plating aliquots of sterilized soil on culture media, and no microbial growth was observed in any of the sterility controls.

Triplicates of the inoculated seeds at each condition were cultivated in 20 L pots in a greenhouse (under natural conditions) trial located at Facultad de Ciencias Agronómicas, Universidad de Chile, in La Pintana, Metropolitan Region of Chile (33°34’10.5"S 70°37’53.1"W). At the initial time of the assay (“Ti”), inoculated seeds were maintained at field-capacity (full irrigation), irrigating three times a week for 120 days. Pots were distributed in a completely random design inside the greenhouse, with 50 cm spacing between rows. One hundred and twenty days after sowing (“das”), all plants were subjected to a drought treatment (initial time of drought treatment; “Tid”) for 12 days. Net photosynthetic rate (Pn) was monitored every other day during this period to determine the progression of drought stress across the plant population. Based on these measurements, the end of the drought period exhibited a reduction of Pn below 50% of their values at Tid. Following drought exposure, plants were re-watered and returned to field-capacity conditions (initial time of post-drought recovery treatment; “Tir”) for 8 days. Pn was similarly monitored to track recovery dynamics, and the final sampling time (“Tf”) was defined when approximately 50% of plants recovered to more than 70% of their Pn values at Tid. Leaf relative water content (RWC) and stem water potential (Ψ_stem_) were measured in all plants at Tid and Tf.

Pn was assessed employing an open portable photosynthesis system Li-6400 (LI-COR Biosciences) equipped with a LI-6400‐40 Leaf Chamber Fluorometer. Measurements were performed on fully sun‐exposed and extended leaves. During these measurements, air CO_2_ concentration was controlled using the injection system and compressed CO_2_ cylinders with a CO_2_ concentration of 380 ppm. Measurements were done at a saturating PAR light intensity, between 11:00 and 13:00 h, which is the period of maximum photosynthetic rates. The area of the leaves was measured to correct the LICOR outputs. Relative water content from all plants was determined by weighing the leaf fresh weight, leaf dry weight, and saturated weight after 18 h floating of leaves on water using the formula described by Anjum et al. (2016) [43]. The leaves’ dry weight was obtained by oven drying at 70 °C for 72 h. Ψ_stem_ measurements were performed in adult mature and fully expanded non-transpiring leaves directly connected to the main stem, which had been bagged with both plastic sheet and aluminum foil at least one hour before measurement [44]. Bagging prevented leaf transpiration, so leaf water potential equaled stem water potential [45]. Each bagged leaf was then cut from the plant and placed inside a pressure chamber (model 1505D, PMS Instrument) for measurement.

**Fig. 1:**
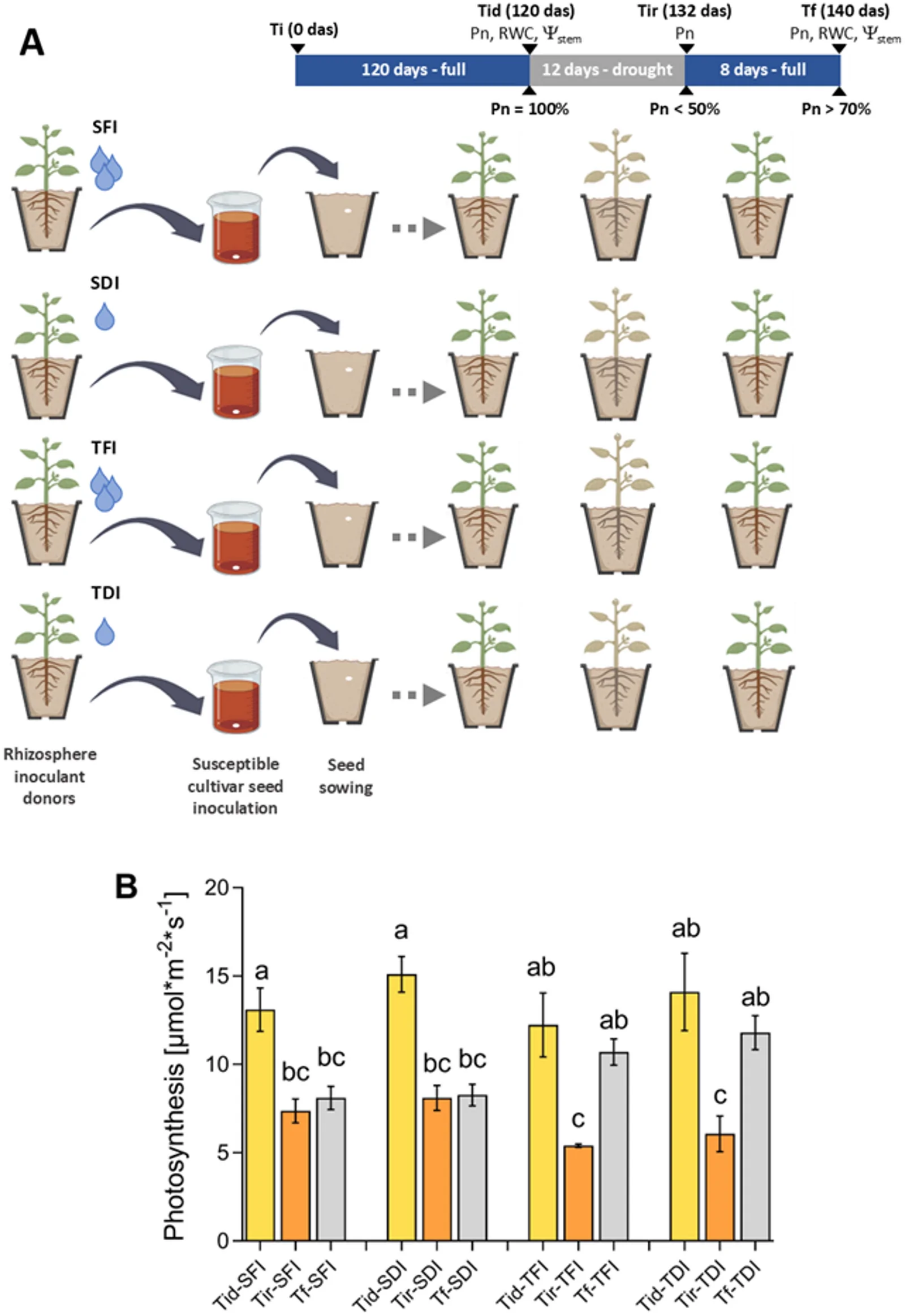
Experimental design and photosynthetic response of tomato plants. (**A**) Schematic overview of the experimental design. At the initial time of the assay (Ti, 0 days, samples correspond to the donor rhizosphere inocula collected immediately before transplantation to recipient plants), seeds of a water-deficit–susceptible cv. Advantage were independently inoculated with rhizosphere communities obtained from four donor treatments: SFI, SDI, TFI, and TDI. Each treatment was established in triplicate. Tid was imposed at 120 das and maintained for 12 days, followed by a post-drought recovery period starting at 132 das (Tir) and ending at 140 das (Tf). RWC and Ψ_stem_ were measured at Tid and Tf, Pn was monitored every other day from Tid to Tf (for details see Methods section). The image was created using BioRender. (**B**) Pn of plants inoculated with different rhizosphere communities during drought and post-drought recovery. Photosynthetic responses were used to assess the contribution of each rhizosphere inoculant to plant drought tolerance and to define the Tir and Tf sampling points. Data are presented as mean ± SE. Tid, Tir, and Tf are shown as yellow, orange, and grey bars, respectively. Different letters indicate significant differences among treatments (Kruskal–Wallis test, *p* < 0.05, followed by Dunn’s post hoc test). Complete physiological data (mean ± SE) are provided in Additional file 1

The trial was daily inspected, and pests and diseases were controlled promptly, while weeds were controlled manually as soon as they were detected. Plant physiological measurements at Tid and Tf were compared using the three replicates per plant and measuring the parameters three times in each plant in a Kruskal–Wallis one-way analysis of variance and Dunn’s post-hoc test. Differences between the groups were considered significant if *p* was < 0.05. The statistical analyses were done using GraphPad Prism 10 software. A scheme of the assay’s design is displayed in Fig. 1A.

### Rhizosphere sampling, DNA extraction and sequencing

At Tf (140 das), rhizospheres from the plants used in this study (*n* = 12) were collected, and their DNA was extracted and stored at -20 °C until sequencing. Rhizosphere collection, DNA extraction and sequencing were performed using the same methodology described in Gaete et al. (2021) [40], thereby ensuring methodological consistency between Ti and Tf datasets.

DNA from each soil sample was extracted using the Qiagen kit DNeasy Blood & Tissue combined with the CTAB based method [46–48]. Briefly, 5 g of soil were resuspended in 5 mL extraction buffer [100 mM Tris-HCl; pH 8, 100 mM Na EDTA; pH 8, 100 mM Na_2_HPO_4_, 1.5 M NaCl, 1% (w/v CTAB] and then 10 mg/mL (final concentration) of lysozyme and 10 µL of Proteinase K (20 mg/mL) were added and mixed by vortex, followed by incubation at 65 °C for 2 h with constant mixing. The mixture was centrifuged at 2000 × *g* for 5 min at RT, and the supernatant fluid was transferred to a clean tube and mixed by vortex with 1 mL of Qiagen AL Binding Buffer and 1 mL of ethanol 100%. Themixture was transferred into the DNeasy mini spin column to continue the kit protocol. Following extraction, DNA samples were further purified using the Qiagen DNeasy PowerClean Pro Cleanup Kit according to the manufacturer’s instructions to remove potential PCR inhibitors commonly present in soil samples. DNA purity was assessed spectrophotometrically, and A260/A280 ratios ranged from 1.7 to 2.1. DNA integrity was evaluated by electrophoresis in Agilent 2200 TapeStation, and only good quality DNA was selected for sequencing. DNA concentration was measured by fluorescence using the Qubit dsDNA HS Assay Kit. Then, DNA was stored at 4 °C until use.

To monitor potential contamination during laboratory procedures, negative controls were included throughout the workflow. DNA extraction negative controls were included by performing the complete extraction protocol in the absence of biological material. All controls were processed using the same reagents and conditions as the environmental samples. Extraction blanks did not yield detectable DNA, and no amplification was observed in PCR negative controls when tested with bacterial and fungal primer sets.

Microbial DNA was amplified using the bacteria-specific primer set, 28 F (5′-GA GTT TGA TCM TGG CTC AG-3′) [49] and 519 R (5′-GWA TTA CCG CGG CKG CTG-3′) [50], flanking variable regions V1-V3 of the 16S rRNA gene, and ITS1 (5′-TCC GTA GGT GAA CCT GCGG-3′) and ITS2 (5′-GCT GCG TTC TTC ATC GAT GC-3′), flanking fungal ITS1 variable region [51]. Amplification was performed using the Qiagen Kit HotStarTaq Plus Master Mix under the following conditions: initial denaturation at 94 °C for 3 min followed by 28 cycles, each set at 94 °C for 30 s, 53 °C for 40 s, and 72 °C for 1 min, with a final elongation step at 72 °C for 5 min. PCR products were used to prepare DNA libraries following Illumina TruSeq DNA library preparation protocol. Sequencings were performed at the Molecular Research DNA laboratory (Shallowater, TX, USA) on an Illumina MiSeq platform in an overlapping 2 × 300 bp configuration to obtain a minimum throughput of 40,000 sequences (reads) per sample.

### Processing of Illumina sequence data and taxonomic identification

Raw amplicon sequences from the donor rhizosphere inoculants (samples at Ti from Gaete et al. 2021 [40]) and from the rhizospheres obtained from this study at Tf, were processed and analyzed with QIIME 2 2019.10 [52]. First, overlapping paired-end reads were joined and depleted of barcodes. Raw sequence data were quality filtered with the minimal quality median set to 30 using the q2-demux plugin followed by denoising with DADA2 [53] (via q2-dada2). Taxonomy was assigned to amplicon sequence variants (ASVs) using the q2-feature-classifier [54] classify-sklearn naïve Bayes taxonomy classifier against the SILVA database (version 138) for bacteria and UNITE database (version 8.0) for fungi. Sequences other than bacteria and fungi were removed. By doing this, we obtained 6,549 bacterial ASVs and 2,066 fungal ASVs. For analyses, we selected reads that mapped with ASVs that were identified in at least two out of three replicates of each sample to analyze data using representative ASVs within each sample. Using this criterion, 880 bacterial and 607 fungal ASVs were included in the analyses.

To identify bacterial and fungal families discriminating between sampling periods, Linear Discriminant Analysis Effect Size (LEfSe) was performed (*p* < 0.05) [55], followed by Linear Discriminant Analysis with a minimum LDA score threshold of 2.0.

### Microbial diversity analysis

Alpha diversity metrics (observed ASVs and Shannon index) were estimated using q2-diversity in QIIME2. Rarefaction curves were generated to assess sequencing depth sufficiency. Samples were rarefied to a standardized depth of 18,000 sequences per sample for 16S rRNA data and 28,000 sequences per sample for fungal ITS data. For statistical analyses, each biological replicate (individual pot) was treated as an independent observation (*n* = 3 per condition). Alpha diversity values obtained from the rarefied datasets were compared using an ANOVA test followed by Tukey post hoc test in GraphPad Prism 10.

Beta diversity (Bray-Curtis dissimilarity) between groups was evaluated by a Permutational multivariate analysis of variance (PERMANOVA) using the MicrobiomeAnalyst program [56]. Differences between the groups were considered significant if *p* was < 0.05.

### Predicted functional profiles

The functional groups of bacteria and fungi were inferred by taxonomic assignment of ASVs using the R package MicroEco 2.0.0 [57, 58], querying the FAPROTAX V1.2.12 [59] and FUNGuild V1.1 [60] databases respectively. To identify functional groups discriminating between treatments, LEfSe was performed (*p* < 0.05) [55], followed by a minimum LDA score threshold of 2.0.

### Rhizosphere microbial co-occurrence networks

To generate rhizosphere microbial co-occurrence networks from the microbial communities associated with the inoculants that promoted the highest and lowest post-drought recovery in susceptible tomato plants, ASVs detected in at least three out of the six samples per condition were retained for analysis.

Because microbiome data are compositional by nature, raw ASV counts were first converted to relative abundances and transformed using a centered log-ratio (CLR) transformation after adding a small pseudocount (10^− 6^) to handle zeros. Temporal microbial changes were then calculated as differences between final and initial CLR-transformed abundances (ΔCLR=CLR_Tf-CLR_Ti), which were used as input for network inference. This approach was adopted to minimize biases associated with differences in initial community composition while accounting for compositional effects.

Significant positive and negative associations among ASVs were inferred using the CoNet Cytoscape plug-in method [61]. Associations were evaluated using an ensemble approach combining Pearson and Spearman correlations and Bray–Curtis and Kullback–Leibler dissimilarity measures. For each metric and edge, 1,000 renormalized permutation and bootstrap iterations were performed to assess edge significance and robustness [62]. P-values were corrected for multiple testing using the Benjamini–Hochberg false discovery rate (FDR) correction, and only edges passing the adjusted significance threshold (FDR < 0.05) were retained. Co-occurrence network models were visualized and analyzed in Cytoscape [63].

## Results

### Rhizosphere microbial transplants and plant drought tolerance and post-drought recovery

Building on our previous characterization of rhizosphere microbiomes associated with *Solanum lycopersicum* cultivars with contrasting water-deficit tolerance [40], we investigated whether the origin of the rhizosphere microbiota, defined by the donor cultivar’s water-deficit-tolerance and irrigation regime, affects plant post-drought recovery. Four rhizosphere donor treatments were established: SFI, SDI, TFI, and TDI, and used to inoculate seeds of the susceptible recipient cv. Advantage (for details, see Methods and Fig. 1A).

Plant drought tolerance and post-drought recovery were assessed by measuring Pn at Tid, Tir, and Tf (Fig. 1B; Additional file 1). At Tid, no significant differences in Pn were observed among treatments (*p* > 0.05), indicating that the origin of the donor rhizosphere did not affect plant physiological performance under full irrigation. After 12 days of water deficit (Tir), all plants exhibited a significant reduction in Pn (*p* < 0.05), signifying that drought stress similarly affected all treatments, regardless of the inoculated rhizosphere origin. In contrast, clear differences emerged at the end of the post-drought recovery period (Tf). Only plants inoculated with rhizosphere communities derived from the water-deficit‐tolerant cultivar showed a significant recovery of photosynthetic activity (*p* < 0.05), whereas plants inoculated with rhizospheres from the susceptible cultivar remained impaired. These results indicate that post‐drought recovery measured by Pn, was associated with the water-deficit‐tolerance of the donor cultivar but was independent of its irrigation regime.

### Diversity and taxonomic and functional composition of rhizosphere microbial communities

To assess changes in rhizosphere microbiomes used for microbial transplants during the experiment and to identify taxa potentially involved in post-drought recovery, we characterized bacterial and fungal communities from all rhizosphere samples at Ti and Tf using high-throughput metabarcoding sequencing (HTS). For bacteria, 2,338,535 raw reads were obtained, 924,471 were retained after quality filtering, yielding 6,549 ASVs prior to prevalence filtering and 880 ASVs after prevalence filtering used in downstream analyses. For fungi, 2,554,243 raw reads were obtained, 2,309,125 were retained after quality filtering, yielding 2,066 ASVs prior to prevalence filtering and 607 ASVs used in downstream analyses. Samples were subsequently rarefied to 18,000 sequences per sample for bacteria and 28,000 for fungi prior to diversity analyses (Additional file 2).

**Fig. 2:**
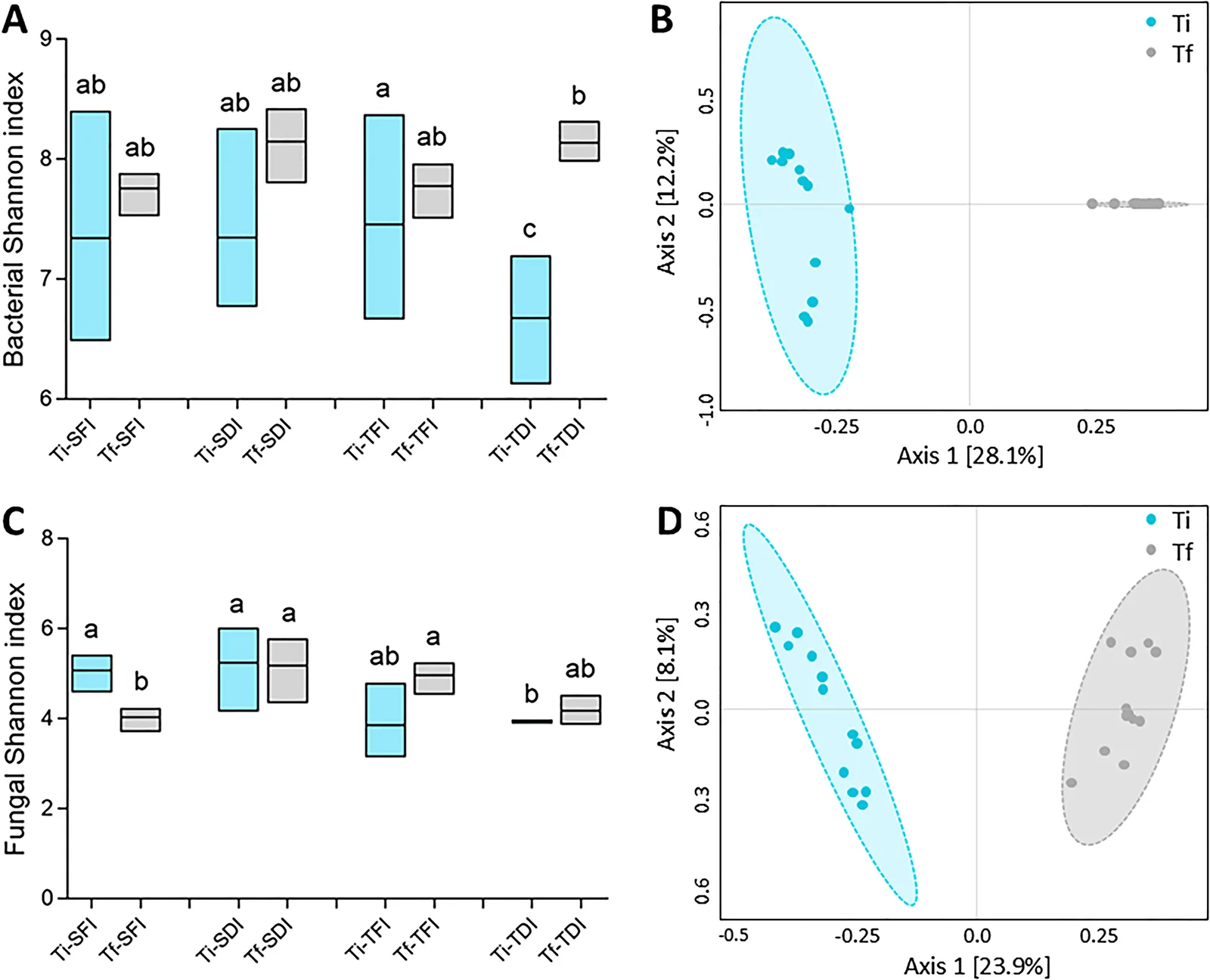
Alpha and beta diversity of rhizosphere microbial communities. **A**, **C.** Bacterial (**A**) and fungal (**C**) alpha diversity estimated using the Shannon index. Alpha diversity was calculated using the three biological replicates per treatment. In the floating bars, horizontal lines indicate the median. Ti corresponds to rhizosphere samples at the initial time of the assay, while Tf corresponds to rhizosphere samples at the final time of the assay. Treatments indicate the origin of the donor rhizosphere: SFI, SDI, TFI, and TDI (for details see Methods section). Different letters indicate statistically significant differences based on comparisons among all groups shown in each panel (ANOVA, *p* < 0.05, followed by Tukey’s post hoc test), including comparisons across sampling times (Ti versus Tf) and among rhizosphere treatments. **B**, **D**. Ordination of bacterial (**B**) and fungal (**D**) community composition based on Bray-Curtis dissimilarities, visualized by principal coordinates analysis (PCoA), with samples grouped by sampling time only (Ti and Tf), pooling all four donor treatments (SFI, SDI, TFI, and TDI). Ellipses indicate clustering of samples by time point

Alpha diversity analyses showed that bacterial Shannon diversity and richness (observed ASVs) were significantly higher from Ti to Tf only for the tolerant inoculant under deficit irrigation (TDI) (*p* < 0.05). In addition, bacterial Shannon diversity in TDI at Ti was significantly lower than in the other inoculants at Ti (*p* < 0.05), whereas at Tf, it reached values comparable to those observed in the other treatments (Fig. 2A and Additional file 3A). In terms of bacterial community composition, beta diversity analyses revealed a clear separation between bacterial communities at the beginning (Ti) and at the end (Tf) of the experiment (PERMANOVA: F = 8.60, R² = 0.281, *p* < 0.001; Fig. 2B).

For fungi, the Shannon index differed significantly between Ti and Tf in rhizospheres from plants grown under full irrigation but only in the susceptible cultivar (Fig. 2C), while richness (observed ASVs) remained stable over time in all rhizosphere treatments (Additional file 3B). Similar to the bacterial communities, fungal beta diversity showed a clear separation between Ti and Tf samples (PERMANOVA: F = 6.66, R² = 0.232, *p* < 0.001; Fig. 2D), indicating that temporal dynamics had a strong effect on rhizosphere microbial community structure, independent of the donor cultivar or irrigation regime.

RWC and Ψ_stem_ were further evaluated to assess plant post-drought recovery (Additional files 1 and 4). Plants inoculated with microbial transplants derived from rhizospheres of fully irrigated plants (SFI and TFI) showed a significant decrease at Tf compared with Tid. In contrast, for Ψ_stem_, only plants inoculated with SFI exhibited a significant difference between Tid and Tf. Collectively, the three physiological measurements (Pn, RWC, and Ψ_stem_) consistently indicated that plants inoculated with TDI exhibited the strongest post-drought recovery, whereas plants inoculated with SFI showed the weakest post-drought recovery response (Fig. 1B and Additional files 1 and 4). In contrast, plants inoculated with SDI and TFI displayed intermediate physiological responses, with significant differences between Tid and Tf observed in only one of the three parameters measured. Based on these results, subsequent microbial analyses focused on comparing rhizosphere communities associated with the donor inoculants that produced the highest (TDI) and lowest (SFI) levels of plant post-drought recovery to maximize the detection of microbial features linked to this trait.

**Fig. 3:**
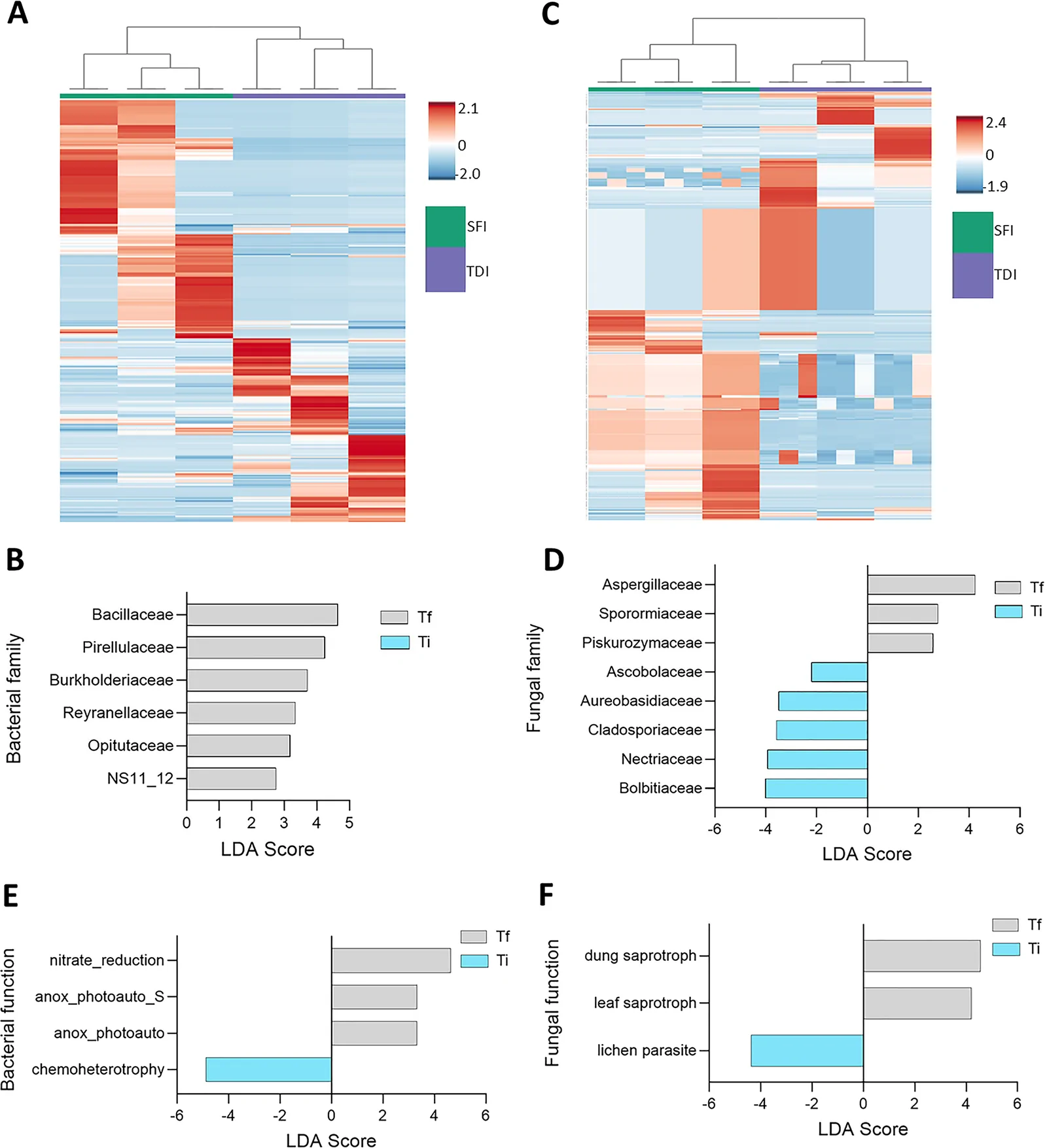
Relative abundance changes and differentially abundant microbial families in SFI and TDI rhizospheres. **A**, **C**. Hierarchical clustering analysis of bacterial (**A**) and fungal (**C**) taxonomic composition based on Euclidean distance using Tf/Ti ratios of ASV relative abundances. Ti corresponds to the initial time of the assay (donor inoculants used for seed inoculation), and Tf corresponds to the final time of the assay. **B**, **D**. LEfSe identifying bacterial (**B**) and fungal (**D**) families that significantly discriminate between Ti and Tf within the TDI rhizosphere condition. Only families identified as significant discriminant biomarkers in TDI and not detected as significant biomarkers in the corresponding SFI comparison are shown. Complete LEfSe results for both TDI and SFI treatments are provided in Additional file 5. **E**, **F**. LEfSe identifying bacterial functions inferred by FAPROTAX **(E)** and fungal trophic guilds inferred by FUNGuild (**F**) that significantly discriminate between Ti and Tf within the TDI rhizosphere condition. ‘anox_photoauto_S’ corresponds to anoxygenic photoautotrophy sulfur-oxidizing. Only functions identified as significant discriminant biomarkers in TDI and not detected as significant biomarkers in the corresponding SFI comparison are shown. Complete LEfSe results for both TDI and SFI treatments are provided in Additional file 6

Given the differences observed in bacterial and fungal diversity at Ti between SFI and TDI, we assessed temporal changes in microbial taxa by calculating the Tf/Ti ratio of ASV relative abundances within each condition. This approach allowed us to account for initial differences in community composition between the SFI and TDI rhizosphere microbiomes. The results shown in Fig. 3A and C indicate that these temporal changes in bacterial and fungal community composition formed distinct clusters for the SFI and TDI conditions in the hierarchical clustering analysis, highlighting the influence of the donor rhizosphere origin (cultivar type and irrigation regime).

Based on these observations, we next sought to identify bacterial and fungal taxa specifically associated with post-drought recovery. To do so, we performed a LEfSe analysis comparing Tf and Ti within the TDI treatment and focused on taxa that discriminated these stages but were not identified as biomarkers in the corresponding SFI comparison. The analysis revealed six bacterial families significantly enriched at Tf relative to Ti in the TDI rhizosphere: *Bacillaceae*, *Pirellulaceae*, *Burkholderiaceae*, *Reyranellaceae*, *Opitutaceae*, and NS11-12, ranked from highest to lowest LDA score (Fig. 3B). No bacterial families were identified as discriminant markers at Ti, suggesting that the post-drought recovery phase was associated with the enrichment of specific bacterial taxa rather than by the discrimination of taxa characteristic of the initial community. In contrast, fungal communities exhibited discriminant markers at both sampling times (Fig. 3D). Three fungal families were enriched at Tf and therefore associated with the post-drought recovery stage (*Aspergillaceae*, *Sporormiaceae*, and *Piskurozymaceae*, ranked from highest to lowest LDA score). Conversely, five fungal families were identified as discriminant at Ti (*Ascobolaceae*, *Aureobasidiaceae*, *Cladosporiaceae*, *Nectriaceae*, and *Bolbitiaceae*), indicating that these taxa were more characteristic of the initial rhizosphere community prior to the post-drought recovery phase. Complete LEfSe results for both TDI and SFI treatments are provided in Additional file 5.

To further characterize the functional potential of the rhizosphere microbiome associated with post-drought recovery, we performed functional annotation of bacterial communities using FAPROTAX and ecological guild assignment of fungal communities using FUNGuild, followed by LEfSe analysis of the resulting functional profiles. FAPROTAX assigned 72.2% of the bacterial ASVs (635 of 880) to a functional group, while FUNGuild assigned ecological guilds to 43.3% of the fungal ASVs (263 of 607).

LEfSe analysis of bacterial functional profiles revealed four functions that discriminated between Ti and Tf in the TDI rhizosphere and were not detected as significant biomarkers in SFI (Fig. 3E). Three functions were enriched at Tf relative to Ti: nitrate reduction, anoxygenic photoautotrophy sulfur-oxidizing (anox_photoauto_S), and anoxygenic photoautotrophy (anox_photoauto), ranked from highest to lowest LDA score. In contrast, chemoheterotrophy was identified as a discriminant function at Ti, indicating it was more characteristic of the initial rhizosphere community prior to the post-drought recovery phase.

LEfSe analysis of fungal guild profiles identified three discriminant guilds in TDI that were absent as significant biomarkers in SFI (Fig. 3F). Two guilds were enriched at Tf and therefore associated with the post-drought recovery stage: dung saprotroph and leaf saprotroph, ranked from highest to lowest LDA score. Conversely, lichen parasite was identified as a discriminant guild at Ti, suggesting it was more representative of the initial fungal community rather than the post-drought recovery phase. Complete LEfSe function results for both TDI and SFI treatments are provided in Additional file 6.

Collectively, these results suggest a marked temporal restructuring of the rhizosphere microbiome under the TDI condition, characterized by the enrichment of specific bacterial and fungal taxa and functions during the post-drought recovery stage. These microbial groups and guilds may therefore contribute to the enhanced post-drought recovery observed in the susceptible tomato cultivar inoculated with the TDI rhizosphere microbiome.

### Co-occurrence network analysis of rhizosphere microbial communities

**Fig. 4:**
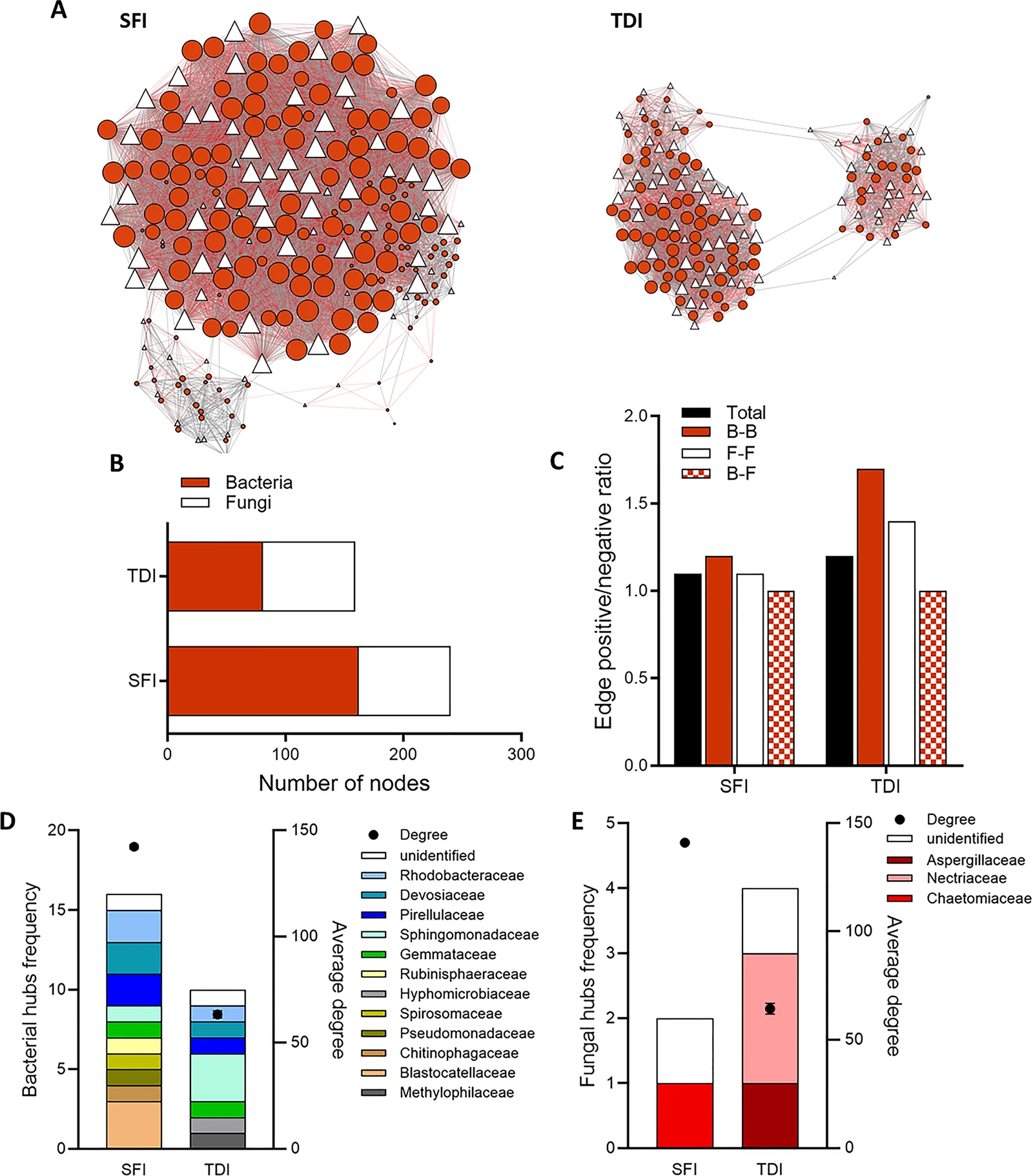
Rhizosphere microbial co-occurrence networks. (**A**) Co-occurrence networks including bacterial (red circles) and fungal (white triangles) ASVs. Interactions were inferred using the ratio of ASV relative abundances between the final time of the assay (end of post-drought recovery treatment; Tf) and the initial time of the assay (donor inoculants used for seed inoculation; Ti). Each node represents an ASV and each edge represents a significant pairwise association (gray lines: positive interactions; red lines: negative interactions). Node size is proportional to node degree (number of connections). Node color and shape indicate microbial kingdom and are intended to facilitate visualization of overall network architecture and interaction patterns rather than identification of individual ASVs. The maximum node degree was 144 for the SFI network and 67 for the TDI network. (**B**) Number of bacterial (red) and fungal (white) nodes (ASVs) included in each co-occurrence network. (**C**) Ratio of positive to negative edges in the networks, including total interactions and those between bacteria-bacteria (B-B), fungi–fungi (F-F), and bacteria-fungi (B-F). **D**-**E**. Taxonomic affiliation (family level; colors) and frequency of hub nodes (defined as ASVs with a degree within the top 13% of the degree distribution in each network; 18 hubs in SFI and 14 hubs in TDI). The black dot indicates the average degree of hub nodes in each network

Because bacterial and fungal taxa potentially involved in post-drought recovery in the susceptible tomato cultivar are unlikely to act in isolation, we performed co-occurrence network analyses to explore their associations with other members of the rhizosphere microbiome (Fig. 4; Additional file 7). To compare microbial interactions between the donor inoculant that promoted the highest plant post-drought recovery (TDI) and the inoculant that produced the lowest post-drought recovery (SFI), while minimizing biases associated with differences in initial community composition, network inference was based on ASV abundance ratios between the final (Tf) and initial (Ti) time points.

The networks exhibited clear structural differences (Fig. 4A; Additional file 7). The SFI network was larger and more densely connected than the TDI network, with 240 and 159 nodes and 10,101 and 3,384 edges, respectively, and a higher average degree (84 vs. 43) and density (0.352 vs. 0.269). Despite similar clustering coefficients (SFI = 0.808; TDI = 0.791), community clustering analysis revealed a more compartmentalized organization in the TDI network (3 clusters) compared with the SFI network (2 clusters), consistent with its lower overall connectivity. The taxonomic composition of the networks also differed markedly. The proportion of fungal nodes was substantially higher in the TDI network (78/159; 49%) than in the SFI network (78/240; 33%) (Fig. 4B).

Analysis of interaction types revealed additional differences between networks (Fig. 4C). In both networks, the ratio of positive to negative edges for bacteria-fungi interactions was lower than the overall community ratio, whereas bacteria-bacteria interactions consistently displayed higher positive-to-negative ratios. Notably, only the TDI network showed a higher positive-to-negative ratio for fungi-fungi interactions than the overall community ratio.

These results suggest that relative to the overall community in both networks, bacteria-fungi interactions are characterized by a lower proportion of positive associations, whereas intra-bacterial associations show a comparatively higher proportion of positive associations. In addition, the rhizosphere associated with the highest plant post-drought recovery (TDI) exhibited a greater prevalence of positive fungal-fungal associations.

To further characterize network organization, we identified hub nodes (defined as the 13% of ASVs with the highest degree; Fig. 4D-E), which represent highly connected taxa that may play central roles in network structure. Hub nodes in the SFI network displayed higher average degrees than those in the TDI network. In total, we detected 16 bacterial and 2 fungal hubs in the SFI network, compared with 10 bacterial and 4 fungal hubs in the TDI network. Bacterial hub taxa were largely shared between both networks; however, *Hyphomicrobiaceae* and *Methylophilaceae* were exclusively detected as hubs in the TDI network. Regarding fungal hubs, many belonged to unidentified families in both networks. *Aspergillaceae* was identified as a hub only in the TDI network, and notably, it was the only fungal hub family that was also identified as significantly enriched at Tf relative to Ti by LEfSe analysis. This suggests a potential role of *Aspergillaceae* in structuring rhizosphere microbial interactions associated with improved post-drought recovery. In contrast, *Nectriaceae*, also detected as a fungal hub exclusively in TDI, was identified by LEfSe as a discriminant taxon at Ti rather than Tf; therefore, it was not considered a candidate taxon linked to the post-drought recovery phase.

## Discussion

Rhizospheres from water-deficit-tolerant and susceptible tomato cultivars, previously exposed or not to water deficit, were used as microbial inoculants to evaluate whether rhizosphere microbiota influence plant post-drought recovery and, if so, which microbial components and interactions may explain this outcome. We specifically focused on the post-drought recovery phase because, in natural and agricultural ecosystems, drought events are often transient rather than permanent, and plants may resume growth or even exhibit compensatory growth following rewatering [64]. Thus, evaluating post-drought recovery provides a more realistic framework to understand plant resilience under climate change scenarios and its implications for crop productivity.

Because all plants were grown in the same sterile soil, differences in soil properties or residual microbial DNA cannot explain the observed changes in microbial communities from Ti to Tf. Consequently, the differential reshaping of the rhizosphere microbiota observed at the end of the experiment depended exclusively on the microbial composition of each donor inoculants, their ability to colonize the rhizosphere, and their interactions within the microbial community. By keeping plant genotype (a single susceptible cultivar) and irrigation regime constant across treatments, both well-known drivers of microbiome assembly [37, 38, 65], we were able to isolate the specific contribution of the microbiota to plant post-drought recovery.

Previous strategies to mitigate environmental stress in crops have largely relied on single plant PGP microorganisms [13, 14] or, more recently, SynComs [66, 67]. However, these approaches often show inconsistent performance outside in vitro, as they fail to capture the ecological complexity of plant-microbe and microbe-microbe interactions that underpin stress tolerance [25]. In this context, microbial transplants derived from naturally assembled rhizosphere communities represent a promising alternative, as they preserve community-level interactions and functionality [36].

Supporting this view, several studies have demonstrated that rhizosphere microbiomes shaped by prior water deficit exposure can enhance plant performance in subsequent hosts, establishing the concept of microbial legacy effects. Lau and Lennon (2012) showed in *Brassica rapa* that plant fitness responses to drought were governed more by the history of the belowground microbial community than by the evolutionary history of the plant itself [35]. Similarly, Monohon et al. (2021) demonstrated in tomato that rhizobacterial communities conditioned under drought stress significantly altered plant biomass and community composition [34], while Carter et al. (2023) showed in maize that a single generation of water-limited microbial conditioning was sufficient to generate a legacy influencing root architecture and photosynthetic function in a subsequent plant cohort [68]. Together with the well-established role of host genotype in shaping rhizosphere microbiome assembly [37, 38, 40], these findings provided the rationale for our dual-legacy hypothesis that also considered the water-deficit-tolerance of the donor plant as an independent legacy component, a gap in the other studies that our research directly addresses.

Our results demonstrate that irrigation history alone is insufficient to explain the magnitude of post-drought recovery observed: TFI and SDI treatments, each carrying only one of the two legacy components, produced intermediate recovery outcomes, whereas TDI, combining both a tolerant donor cultivar and a water-deficit irrigation history, produced the strongest recovery, and SFI, lacking both, produced the weakest (Fig. 1B; Additional files 1 and 4). This synergistic interaction between donor cultivar identity and irrigation history constitutes the dual legacy framework proposed here and cannot be captured when only one source of microbial history is considered.

At the community level, time strongly influenced both bacterial and fungal assemblages, as evidenced by beta diversity patterns (Figs. 2B and D). Nevertheless, significant differences in alpha diversity were already present among donor rhizosphere inoculants at Ti (Fig. 2A and C; Additional file 3), particularly for bacterial communities from the tolerant cultivar in deficit irrigation (TDI) treatment. To account for this initial variability, analyses were normalized relative to Ti abundances.

Hierarchical clustering of temporal changes in ASV relative abundances revealed clear separation between the SFI and TDI conditions (Figs. 3A and C). This pattern indicates that the rhizosphere microbiomes followed distinct restructuring trajectories during post-drought recovery depending on the origin of the donor microbiome. Such divergence supports the concept of a dual legacy effect, in which both the water-deficit-tolerance phenotype of the donor cultivar and its prior irrigation regime shape microbial community responses after transplantation under the controlled conditions of this experiment.

Moreover, the differential enrichment of bacterial and fungal families (Figs. 3B and D) further indicates that specific microbial lineages may contribute to the restructuring of rhizosphere microbiomes shaped by the combined legacy of donor cultivar and irrigation history during plant post-drought recovery. In particular, members of *Bacillaceae* and *Burkholderiaceae* include well-known plant-associated bacteria with documented roles in plant growth promotion, stress tolerance, and suppression of plant pathogens [69–71]. In addition, taxa belonging to *Pirellulaceae*, *Reyranellaceae*, and *Opitutaceae* are commonly detected in soil and rhizosphere environments and are thought to contribute to nutrient cycling and the degradation of complex organic compounds [72–75]. Although their direct effects on plant physiology remain less well characterized, these taxa may influence plant performance indirectly by shaping rhizosphere nutrient availability and microbial community structure. Interestingly, NS11-12 was identified among the bacterial taxa enriched in our analysis. Although members of this group are commonly associated with marine environments, their ecology remains poorly understood, and related lineages have also been detected in non-marine habitats [76, 77]. Further work is required to determine their origin and potential ecological role in the tomato rhizosphere.

Fungal taxa enriched during post-drought recovery may also contribute to these processes. In particular, members of *Aspergillaceae*, *Sporormiaceae*, and *Piskurozymaceae* have been reported in soil and plant-associated environments and are known for their capacity to degrade complex organic substrates and participate in nutrient turnover [78–80]. Notably, members of *Aspergillaceae* have also been reported to enhance drought tolerance across different plant species [81–83]. Such metabolic capabilities may support rhizosphere functioning during plant post-drought recovery by facilitating organic matter decomposition and nutrient availability. Collectively, these findings suggest that post-drought recovery in susceptible tomato plants may be linked not to a single microbial taxon but to a consortium of bacteria and fungi with complementary ecological functions within the rhizosphere microbiome.

Functional annotation of the bacterial communities using FAPROTAX further revealed that the enrichment of specific functional guilds accompanied the taxonomic changes observed in the TDI rhizosphere during post-drought recovery (Fig. 3E). Three bacterial functions were enriched at Tf in TDI: nitrate reduction, anoxygenic photoautotrophy sulfur-oxidizing, and anoxygenic photoautotrophy. The enrichment of nitrate reduction at the end of the recovery phase suggests an increase in nitrogen transformation activity in the TDI rhizosphere, which may contribute to greater nitrogen availability for the recovering plant. Microbial-driven nitrogen transformation in the rhizosphere has been increasingly recognized as a key mechanism through which rhizosphere bacteria support plant growth and stress resilience, with nitrate availability being directly linked to improved photosynthetic performance and biomass recovery under water-limited conditions [84]. The enrichment of anoxygenic photoautotrophic functions at Tf, including sulfur-oxidizing taxa, further indicates shifts in rhizosphere redox chemistry during post-drought recovery. These bacteria, which couple light-driven carbon fixation with reduced sulfur compound oxidation, are known to contribute to sulfur cycling in soil environments and may modulate the availability of sulfur-containing compounds that influence plant metabolism [85].

Ecological guild analysis of fungal communities using FUNGuild revealed that two saprotrophic guilds, dung saprotroph and leaf saprotroph, were enriched at Tf in the TDI rhizosphere (Fig. 3F). Saprotrophic fungi play a fundamental role in the decomposition of complex organic substrates and the release of carbon and nutrients into the soil [86]. Their enrichment during post-drought recovery in TDI suggests an increase in organic matter decomposition activity in the rhizosphere, potentially facilitating nutrient availability for the recovering plant. The enrichment of both dung and leaf saprotrophic guilds indicates functional complementarity in substrate use, potentially enabling more complete organic matter turnover in the TDI rhizosphere. Together, the functional profiles of both bacterial and fungal communities reinforce the view that post-drought recovery in susceptible tomato plants is associated with a rhizosphere microbiome shift toward increased nutrient cycling and metabolic activity, involving complementary bacterial and fungal functional guilds.

Additionally, the functional profiles identified at Tf in TDI show a notable correspondence with the taxonomic biomarkers enriched at the same time point, providing cross-method support for the functional interpretation of the community shifts observed during post-drought recovery. Among bacterial families, *Bacillaceae* and *Burkholderiaceae* are documented contributors to nitrate transformation and denitrification in soil environments [87, 88], consistent with the enrichment of nitrate reduction detected at Tf. For fungal communities, the taxon-function correspondence is particularly clear. *Sporormiaceae* are coprophilous ascomycetes with well-characterized saprotrophic capabilities [89], directly consistent with the dung saprotroph guild enriched at Tf. *Aspergillaceae*, enriched at Tf, includes genera known as efficient decomposers of plant biomass in rhizosphere environments [90], consistent with both the leaf saprotroph and dung saprotroph guilds detected at the same time point in TDI.

To disentangle microbiome-mediated effects beyond changes in relative abundance, we constructed microbial co-occurrence networks using rhizosphere inoculants associated with the highest (TDI) and lowest (SFI) levels of plant post-drought recovery (Fig. 4; Additional file 7). This approach allowed us to evaluate patterns of microbial co-occurrence associated with contrasting post-drought recovery outcomes, rather than focusing solely on changes in individual taxa. The SFI network was larger and more densely connected, whereas the TDI network exhibited lower overall interaction density but a more compartmentalized organization, with three clusters compared to two in SFI. Higher network modularity has been associated with greater ecosystem resilience, as it allows different microbial groups to fulfil their functional roles even under changing environmental conditions [91]. This pattern is consistent with a more specialized and functionally organized rhizosphere microbiome associated with enhanced post-drought recovery, in which microbial groups may interact more selectively rather than forming a broadly connected community.

Beyond overall network structure, fungal communities assumed a more prominent role within the TDI network. Both the proportion of fungal nodes and the relative contribution of positive fungi-fungi interactions were higher in TDI compared to SFI (Fig. 4B and C). These fundings suggest that fungi may play an important role in community organization during post-drought recovery. Fungal co-occurrence networks have been reported to be more resistant to drought than bacterial networks [92]. Moreover, drought can strengthen co-occurrence networks among functional guilds of rhizosphere fungi, consistent with the stress gradient hypothesis predicting increased positive interactions under stress conditions [93]. Together, these observations suggest that the higher prevalence of positive fungal-fungal associations in TDI may reflect cooperative interactions that contribute to rhizosphere stability during post-drought recovery. However, experimental validation, such as fungal manipulation or co-culture approaches, will be required to determine whether these fungal taxa directly influence bacterial community dynamics and ecosystem recovery.

Although network analyses do not demonstrate causal relationships, the distinct network architecture observed in TDI coincided with the greatest physiological recovery across the three physiological parameters evaluated. This correspondence suggests that a more modular microbial network, together with a greater prevalence of positive fungal interactions, may provide a more resilient microbial environment capable of sustaining rhizosphere functions that support plant physiological recovery following water stress.

To identify which taxa may underpin this network structure, we examined hub nodes as highly connected taxa likely to play central structural roles (Fig. 4D and E). Among bacterial hubs, *Hyphomicrobiaceae* and *Methylophilaceae* were exclusively detected in the TDI network. Both families are well-characterized methylotrophic taxa that are abundant in plant-associated soils, where they actively utilize methanol released as a metabolic by-product of plant growth [94, 95]. *Hyphomicrobiaceae* displays diverse chemo-organotrophic metabolic pathways including sulfide oxidation, metal cycling and denitrification, while both families have documented roles in the metabolism of climatically relevant sulfur compounds such as dimethylsulfide in soil environments [96, 97]. Methylotrophic bacteria have additionally been reported to enhance plant growth under drought conditions [98, 99]. Their exclusive presence as hub taxa in the TDI network suggests that methylotrophic activity may contribute to the functional organization of the rhizosphere microbiome associated with post-drought recovery, potentially through carbon cycling and modulation of volatile compound metabolism, though direct functional evidence remains to be established.

Among fungal hubs, *Aspergillaceae* was exclusively detected in the TDI network and was the only fungal hub family also identified as significantly enriched at Tf by LEfSe analysis, reinforcing its relevance as a candidate taxon linking network structure to post-drought recovery. Members of *Aspergillaceae* have been associated with drought tolerance promotion in plants through multiple mechanisms, including phytohormone production, nutrient mobilization, and stimulation of plant antioxidant defenses [100, 101]. Notably, *Aspergillaceae* was also assigned to saprotrophic guilds in the FUNGuild analysis, suggesting an additional role in organic matter decomposition and nutrient cycling during post-drought recovery. The convergence of evidence from LEfSe enrichment, saprotrophic guild assignment, and network hub status positions *Aspergillaceae* as a priority candidate taxon for future functional validation studies aimed at elucidating its contribution to rhizosphere-mediated post-drought recovery.

Collectively, these results support a “dual legacy” framework, in which both biotic (host tolerance) and abiotic (watering history) factors interact to determine microbiome-mediated plant responses. This extends current models of microbiome inheritance that have primarily emphasized environmental history. From an applied perspective, our findings suggest that rhizosphere pre-conditioning strategies integrating both host plant tolerance traits and prior watering regimes could be leveraged to enhance crop post-drought recovery. For instance, soils could be microbially pre-conditioned using drought-tolerant cultivars exposed to defined stress regimes prior to crop establishment.

Despite the strengths of this experimental design, several limitations should be acknowledged. First, the use of sterilized soil allowed us to isolate microbiome-driven effects with high internal validity and minimize background variation from indigenous microorganisms. However, this approach simplifies the ecological context in which plant-microbe interactions naturally occur and may reduce ecological realism relative to agricultural soils. In natural systems, introduced microorganisms must establish, persist, and interact with resident microbial communities, which may influence both their colonization success and their effects on plant post-drought recovery. Likewise, greenhouse conditions cannot fully capture the environmental heterogeneity and biotic complexity of field systems. Therefore, future studies should evaluate the stability, colonization success, compatibility with native soil microbiomes, and functional impacts of these transplanted communities in non-sterile soils and under field conditions to assess their applicability in agricultural systems.

Second, although community composition, co-occurrence patterns, and predictive functional analyses provided indirect evidence of potential mechanisms associated with post-drought recovery, direct measurements of microbial activity, such as metabolomics, transcriptomics, or other activity-based approaches, were beyond the scope of this study and will be necessary to elucidate the biochemical processes underlying the observed plant responses.

Third, the relatively low number of biological replicates (*n* = 3 per treatment) may have reduced statistical power to detect subtle differences in microbial diversity, biomarker identification, and limited the robustness of network inference. Consequently, the co-occurrence networks presented here should be considered primarily as an exploratory framework for comparing global interaction patterns between treatments rather than definitive ecological interactions. Thus, even though our results support the existence of microbial dual legacy over the time scale examined in this study, future studies with larger sample sizes and longer experimental time scales will be required to determine the persistence and stability of these legacies across multiple cultivation cycles and to formally assess temporal differences among inoculant types.

Finally, the lack of microbial community characterization at the end of the drought period prevents discrimination between microbiome shifts occurring during drought and those specifically associated with post-drought recovery. This trade-off was necessary to maintain a longitudinal design based on the same individuals and minimize inter-plant variability. Future studies including destructive sampling of parallel plant cohorts and longer-term experiments across multiple cultivation cycles will help resolve microbial dynamics and determine the persistence and stability of drought-associated rhizosphere microbial legacy effects. Nevertheless, our results support the existence of such legacy effects within the temporal scale examined in this study.

## Conclusions

In conclusion, this study provides experimental evidence that the legacy of entire rhizosphere communities from cultivars differing in water-deficit-tolerance and watering history jointly shapes plant post-drought recovery. The concept of a “dual legacy”, integrating host phenotype and environmental history, offers a new framework for understanding microbiome-mediated plant resilience. While demonstrated here in tomato, this framework may be transferable to other crops, stressors (such as salinity or heat), and regenerative agricultural systems that seek to harness naturally assembled microbial communities for climate-resilient food production.

## Supplementary Information

Below is the link to the electronic supplementary material.

Additional file 1

Additional file 2

Additional file 3

Additional file 4

Additional file 5

Additional file 6

Additional file 7

## Data Availability

All sequence data used in this study have been deposited in the Sequence Read Archive (SRA) of the National Center for Biotechnology Information (NCBI) under the BioProject accession number PRJNA1006696.

## Abbreviations

PGP: Plant Growth-Promoting
SynComs: Synthetic Communities
SFI: Susceptible cultivar in Full Irrigation
SDI: Susceptible cultivar in Deficit Irrigation
TFI: Tolerant cultivar in Full Irrigation
TDI: Tolerant cultivar in Deficit Irrigation
Tid: Initial time of drought treatment
Tir: Initial time of post-drought recovery treatment
Tf: Final time of the assay
Pn: Net photosynthetic rate
RWC: Relative water content
Ψstem: Water potential
LEfSe: Linear Discriminant Analysis Effect Size
HTS: High-Throughput metabarcoding Sequencing
