# Supplementary material for: Dual legacy of plant water-deficit-tolerance and watering history shapes rhizosphere microbial transplant outcomes in tomato post-drought recovery
Source: Environ Microbiome. 2026 Sep 8;21:115. doi: 10.1186/s40793-026-00953-0 (PMC13633642; doi:10.1186/s40793-026-00953-0)
Supplement: Supplementary file 1 [file 40793_2026_953_MOESM1_ESM.docx]

**Additional file 1**

**Additional file 1.** Mean ± SE of net photosynthetic rate, leaf relative water content, and stem water potential for each treatment and sampling time. Measurements were performed before drought imposition (Tid), after drought (Tir), and after the post-drought recovery period (Tf).
