# Supplementary material for: Dual legacy of plant water-deficit-tolerance and watering history shapes rhizosphere microbial transplant outcomes in tomato post-drought recovery
Source: Environ Microbiome. 2026 Sep 8;21:115. doi: 10.1186/s40793-026-00953-0 (PMC13633642; doi:10.1186/s40793-026-00953-0)
Supplement: Supplementary file 2 [file 40793_2026_953_MOESM2_ESM.docx]

**Additional file 2**

**Additional file 2. Rarefaction curves of microbial communities based on observed features.** Rarefaction analysis was performed using the bacterial ASV table after sequence quality filtering and denoising. Gray lines represent individual samples, whereas colored lines represent the mean number of observed features (ASVs) for samples collected at the initial time of the assay (Ti; donor inoculants used for seed inoculation; dark gray line) and at the final time of the assay (Tf; rhizosphere samples collected at the end of the post-drought recovery period; light blue line). The curves approached a plateau with increasing sequencing depth, indicating that sequencing effort was sufficient to capture most bacterial (A) and fungal (B) diversity present in the samples.
