# Supplementary material for: Dual legacy of plant water-deficit-tolerance and watering history shapes rhizosphere microbial transplant outcomes in tomato post-drought recovery
Source: Environ Microbiome. 2026 Sep 8;21:115. doi: 10.1186/s40793-026-00953-0 (PMC13633642; doi:10.1186/s40793-026-00953-0)
Supplement: Supplementary file 3 [file 40793_2026_953_MOESM3_ESM.docx]

**Additional file 3**

**Additional file 3. Richness of rhizosphere microbial communities.** A, B. Bacterial (A) and fungal (B) alpha diversity estimated using observed ASV richness. Alpha diversity was calculated using the three biological replicates per treatment. In the floating bars, horizontal lines indicate the median. Ti corresponds to rhizosphere samples at the initial time of the assay (donor rhizosphere inoculants; light blue boxplots), while Tf corresponds to rhizosphere samples at the final time of the assay (grey boxplots). Treatments represent the origin of the donor rhizosphere: SFI (susceptible cultivar under full irrigation), SDI (susceptible cultivar under deficit irrigation), TFI (tolerant cultivar under full irrigation), and TDI (tolerant cultivar under deficit irrigation). A. Different letters indicate statistically significant differences, B. No statistically significant differences were observed among treatments (ANOVA test, p < 0.05, followed by Tukey post hoc test).
