# Supplementary material for: Dual legacy of plant water-deficit-tolerance and watering history shapes rhizosphere microbial transplant outcomes in tomato post-drought recovery
Source: Environ Microbiome. 2026 Sep 8;21:115. doi: 10.1186/s40793-026-00953-0 (PMC13633642; doi:10.1186/s40793-026-00953-0)
Supplement: Supplementary file 4 [file 40793_2026_953_MOESM4_ESM.docx]

**Additional file 4**

**Additional file 4. Plant physiological parameters**. A,B. Leaf relative water content (RWC) (A) and stem water potential (Ψ_stem_) (B) measured in plants to evaluate which rhizosphere inoculants used for seed inoculation promoted plant recovery from drought. Tid corresponds to the initial time of the drought treatment (yellow bars), and Tf corresponds to the final time of the assay (grey bars). Treatments represent the origin of the donor rhizosphere: SFI (susceptible cultivar under full irrigation), SDI (susceptible cultivar under deficit irrigation), TFI (tolerant cultivar under full irrigation), and TDI (tolerant cultivar under deficit irrigation). Data are presented as mean ± SE. Different letters indicate statistically significant differences among treatments (Kruskal–Wallis test, p < 0.05, followed by Dunn’s post hoc test). Complete physiological data (mean ± SE) are provided in Additional file 1.
