# Supplementary material for: Dual legacy of plant water-deficit-tolerance and watering history shapes rhizosphere microbial transplant outcomes in tomato post-drought recovery
Source: Environ Microbiome. 2026 Sep 8;21:115. doi: 10.1186/s40793-026-00953-0 (PMC13633642; doi:10.1186/s40793-026-00953-0)
Supplement: Supplementary file 5 [file 40793_2026_953_MOESM7_ESM.docx]

**Additional file 7**

**Additional file 7.** Networks' parameters.

| **Parameter** | **SFI** | **TDI** |
| --- | --- | --- |
| Number of nodes | 240 | 159 |
| Number of edges | 10,101 | 3,384 |
| Average degree | 84 | 43 |
| Density | 0.352 | 0.269 |
| Clustering coefficient | 0.808 | 0.791 |
| Number of clusters | 2 | 3 |
| Total positive/negative edges | 1.07 | 1.22 |
| Connected components | 1 | 1 |
